# Cross-border comparison of the Dutch and German guidelines on multidrug-resistant Gram-negative microorganisms

**DOI:** 10.1186/s13756-015-0047-6

**Published:** 2015-02-27

**Authors:** Jan Müller, Andreas Voss, Robin Köck, Bhanu Sinha, John W Rossen, Martin Kaase, Martin Mielke, Inka Daniels-Haardt, Annette Jurke, Ron Hendrix, Jan A Kluytmans, Marjolein F Kluytmans-van den Bergh, Matthias Pulz, Jörg Herrmann, Winfried V Kern, Constanze Wendt, Alex W Friedrich

**Affiliations:** Department of Medical Microbiology, University of Groningen, University Medical Center Groningen, Hanzeplein 1, hpc EB80, 9713 GZ Groningen, The Netherlands; Department of Medical Microbiology, Canisius-Wilhelmina Hospital and Radboud University Medical Center, Nijmegen, The Netherlands; Institute of Hygiene, University Hospital Münster, Münster, Germany; National Reference Laboratory for Multidrug Resistant Gram-negative Bacteria, Department of Medical Microbiology, Ruhr University Bochum, Bochum, Germany; Robert Koch-Institute, Berlin, Germany; Division of Health Protection, Health Promotion, NRW Centre for Health, Münster, Germany; Department of Infectiology and Hygiene, NRW Centre for Health, Münster, Germany; Laboratory for Microbiology and Infection Control, Amphia Hospital, Breda, The Netherlands; Amphia Academy Infectious Disease Foundation, Amphia Hospital, Breda, The Netherlands; Governmental Institute of Public Health of Lower Saxony, Hannover, Germany; Institute for Hygiene, University Hospital Oldenburg, Oldenburg, Germany; Center for Infectious Diseases and Travel Medicine, University Hospital Freiburg, Freiburg, Germany; Hygiene-Institute, University of Heidelberg, Heidelberg, Germany

**Keywords:** MDRO, Gram-negative organism, Cross-border healthcare, Infection prevention guidelines

## Abstract

**Background:**

In all European countries, hospital-acquired infections caused by Gram-negative multidrug-resistant microorganisms (GN-MDRO) are a major health threat, as these pathogens cannot be adequately treated anymore, or the start of effective antibiotic treatment is delayed. The efforts to limit the selection and spread of GN-MDRO remains a problem in cross-border healthcare, as the national guidelines on hygiene standards applicable for patients colonized or infected with GN-MDRO in hospitals are not harmonized between European countries.

**Methods:**

In order to point out the similarities and differences in the national guidelines of Germany and The Netherlands regarding GN-MDRO, guidelines were compared and an expert workshop was organized by the INTERREG IVa project EurSafety Health-net.

**Results:**

Both guidelines divide the Gram-negative organisms into subgroups based on bacterial species and antibiotic susceptibility patterns in order to define multidrug-resistant variants of these bacteria. However, the Dutch guideline defines that GN-MDRO *Enterobacteriaceae* requires testing for certain mechanisms causing antibiotic resistance, whereas the German guideline makes use of a newly created classification scheme, based on phenotypic characterization. Besides diagnostic issues, the main difference between the Dutch and German guideline is the divergent evaluation of ESBL-producing *Enterobacteriaceae*. Special hygiene measures are required for all patients with ESBL-producing *Enterobacteriaceae* in The Netherlands, whereas the German guideline recommends special precautions only for those cases in which patients are colonized or infected with strains showing co-resistance to ciprofloxacin (“3MRGN”).

**Conclusions:**

The usage of consistent terminology and harmonized diagnostic procedures would improve the possibilities for infection prevention, treatment and patient safety. Prevention of severe non-treatable infections and outbreaks due to MDRO, caused by an increased population seeking medical treatment abroad together with an increased number of highly susceptible individuals demands gathering of regional data, and data comparable between the two sides of the Dutch-German border. The necessity to cooperate multidisciplinary and across borders is required to prevent a post-antibiotic era – in which common infections and minor injuries may lead to death.

## Background

Gram-negative multidrug-resistant microorganisms (GN-MDRO), such as Extended Spectrum β-Lactamase (ESBL) producing or Carbapenem-resistant *Enterobacteriaceae* and multidrug-resistant *Pseudomonas aeruginosa* and *Acinetobacter baumannii*, cause an incremental part of hospital-acquired infections in all European countries [1,2]. This may result in serious health threats, as relatively harmless infections may become severe diseases, if these pathogens cannot be adequately treated anymore or start of effective antibiotic treatment is delayed [3,4]. Besides direct health threats to the patient, GN-MDRO are responsible for additional negative impacts, such as increased morbidity, increased length of hospital stay and higher costs to the healthcare system [5].

In Europe, it is estimated that about 25,000 people die as a direct result of GN-MRDO infections every year [6]. Hence, protecting patients from these infections is among the most important challenges in European healthcare settings. Preventive measures that can be applied to achieve this aim include antibiotic stewardship, promotion of hand hygiene, consequently implemented standard infection prevention precautions, additional transmission based precautions (e.g. single-rooms) and efforts to limit the selection and spread of GN-MDRO in animals, food and the environment within “One Health”-initiatives.

As international travel and international patient care are considered to represent major risk factors for acquiring GN-MDRO, European countries need to implement measures to prevent transmission of GN-MDRO between healthcare institutions abroad and national facilities [7,8]. Exemplarily, The Netherlands (Dutch Working Party for Infection Control, WIP) and Germany (Commission of Hospital hygiene and Infection prevention, KRINKO) have recently revised their national guidelines on hygiene standards applicable for patients colonized or infected with GN-MDRO in hospitals [9,10].

As these guidelines are not harmonized between countries, definitions, nomenclature of GN-MDRO and the respective precaution measures recommended differ to some respect. For the two countries, which share a common border for 567 km, these differences may lead to special challenges in cross-border patient care. This occurs when patients are treated in hospitals on both sides of the border or are transferred from one country to the other. One of the main goals of this cross-border region is to prevent the spread of especially carbapenemase producing *Enterobacteriaceae*.

This issue is of special importance, because the Directive 2011/24/EU published by the European Union that came into force on October 25th 2013, clearly outlines the patients’ right to seek healthcare service across European borders. In addition, it has already been demonstrated that patient transfers between hospitals significantly increase the transmission of hospital-acquired infections at the regional and national scale [11].

Therefore, in this article, we describe and compare the current Dutch and German GN-MDRO definitions and prevention guidelines for hospitals. The aim of this article is to point out differences between the two national guidelines and the occurrence of MDRO with regard to the epidemiology, prevention measures and diagnostics that need to be considered when cross-border patient care is performed in order to forestall misunderstandings, ensure patient safety and guarantee sustainable healthcare.

## Methods

Primarily, the current versions of the German and Dutch guidelines on GN-MDRO prevention in healthcare facilities were analysed with respect to similarities and differences taking into account definitions, nomenclatures and hygienic precaution measures to be taken by healthcare workers [9,10]. Afterwards, a cross-border workshop on the national GN-MDRO guidelines was organized within the framework of the INTERREG IVa project EurSafety Health-net (www.eursafety.eu) in order to discuss the differences observed between the two national guidelines. About 75 experts in the field of Medical Microbiology and Infection Prevention from Germany and The Netherlands attended the workshop “ESBL/Multidrug Resistant Gram-negatives (MRGN) harmonization of the new guidelines in Germany and The Netherlands” in Nijmegen, The Netherlands, on December 11th, 2013. Stakeholders coming from the cross-border region of Germany and the Netherlands, were able to define their own questions regarding GN-MDRO in the border area. Due to the experts’ different areas of expertise, three dicussion groups were formed. Discussing on the Epidemiology, Infection Prevention or Diagnostics & Treatment of GN-MDRO, the following questions were tried to answer:

i) What are the similarities and differences between the Dutch and German Guideline?
ii) May problems be caused by cross-border patient care?
iii) Which actions are required to harmonize both guidelines?

## Results

Comparison of the two guidelines indicated that the definition of GN-MDRO, which is important for planning specific hygiene measures, differs between the Dutch and the German guidelines. Tables 1 and 2 show a compilation of the definitions used. Both guidelines divide the Gram-negative organisms in subgroups based on bacterial species and antibiotic susceptibility patterns in order to define multidrug-resistant variants of these bacteria (GN-MDRO *sensu stricto*). GN-MDRO are designated “BRMO” (Bijzonder resistente micro-organismen) according to the Dutch guideline and “3MRGN” (Multi-resistente Gramnegative Erreger), e.g. ESBL-producing *K. pneumoniae* with resistance to ciprofloxacin, or “4MRGN”, e.g. resistant to four antibiotic classes or carbapenemase-producing *Enterobacteriaceae*, according to the German guideline.

**Table 1:** **Definition of Gram-negative Multidrug-resistant organisms according to German guideline**

**Antibiotic**	***Enterobacteriaceae, Acinetobacter baumannii***		***Pseudomonas aeruginosa***	
	**3MRGN**	**4MRGN**	**3MRGN**	**4MRGN**
Piperacillin	R	R	Only one antibiotic group is susceptible	R
Cefotaxime/Ceftazidime	R	R		R
Imipenem/meropenem	S	R		R
Ciprofloxacin	R	R		R
Specifications/exceptions	Count as “R” if **one** antibiotic of the category is resistant (e.g. imipenem **OR** meropenem).		Count as “R” if **all** antibiotics of each category are resistant (e.g. imipenem **AND** meropenem).	
	If “R” for imipenem or meropenem, then always report as 4MRGN (irresp. of test result for other antibiotics).		Cefepime can also be considered in addition to ceftazidime.	
	If carbapenemase is detected, always categorize as 4MRGN.			

Definition of Gram-negative Multidrug-resistant organisms (GN-MDRO), designated “MRGN”, is based on results of phenotypic susceptibility testing to four antibiotic classes before the antibiogram is modified by interpretative reading. R = resistant or intermediate; S = susceptible. *Enterobacteriaceae* are categorized as “3MRGN” (GN-MDRO with resistance to 3 antibiotic classes), if carbapenems are still susceptible and “4MRGN” (GN-MDRO with resistance to 4 antibiotic classes), if not. *P. aeruginosa* isolates are categorized as “3MRGN” if at least one of the four antibiotic classes is susceptible and “4MRGN”, if all are resistant. Other pheno- or genotypic test results are not considered for the definition with the important exception of carbapenemase production which automatically lead to a classification as “4MRGN”.

**Table 2:** **Definition of Gram-negative multidrug-resistant organisms according to Dutch guideline**

**Antibiotic, resistance mechanism**	***Enterobacteriaceae***	***Pseudomonas aeruginosa***	***Acinetobacter sp.***	***Stenotrphomonas maltophilia***
Piperacillin		C		
ESBL positive	A			
Ceftazidime		C		
Carbapenemase positive	A	C	A	
Quinolones	B	C	B*	
Aminoglycosides	B	C	B	
Cotrimoxazole				A

Definition of GN-MDRO, designated “BRMO”, is based on results of phenotypic susceptibility testing to four antibiotic classes (piperacillin, ceftazidime, quinolones, aminoglycosides and cotrimoxazole) and resistance mechanisms. **A** = Bacteria have to be resistant against **this one** antibiotic group to fulfil at the definition. **B** = Bacteria have to be resistant against a least **two** of these antibiotic groups to fulfil at the definition. **C** = Bacteria have to be resistant against at least **three of these** antibiotic groups to fulfil at the definition.

*Except norfloxacin.

On species level, these subgroups comprise *Enterobacteriaceae, Acinetobacter* (in the Dutch guideline *Acinetobacter spp.*, in the German guideline only species of the *A. baumannii*-group), and *Pseudomonas aeruginosa*. The Dutch guideline also defines multidrug-resistant variants of *Stenotrophomonas maltophilia.*

Antibiotics that are considered relevant for the definition of multidrug-resistance are similar in both guidelines: piperacillin, cefotaxime, ceftazidime, quinolons (i.e. ciprofloxacin according to German guideline), carbapenems (i.e. meropenem and/or imipenem according to German guideline) or confirmation of carbapenemase production. Additionally, the Dutch guideline also takes into consideration ESBL-phenotype and phenotypic resistance to cotrimoxazole and aminoglycosides. The German guideline additionally considers cefepime in case of *P. aeruginosa*.

If defined as GN-MDRO, hygienic measures, which exceed standard precautions, have to be applied in order to prevent spread in the hospital according to both guidelines. Depending on the type of microorganism, these special precaution measures may differ. This is shown in Table 3.

**Table 3:** **Precaution measures for BRMO and MRGN according to Dutch and German guidelines**

**Species**	**German guideline**				**Dutch guideline**
	**3MRGN**		**4MRGN**		**BRMO**
	High-risk ward*	Normal ward*	High-risk ward*	Normal ward*	**Hospital-wide**
*E. coli*	Single-room	Standard	Single-room	Single-room	Contact; Single-room, if CPE
*Klebsiella sp.*	Single-room	Standard	Single-room	Single-room	Contact; Single-room, if CPE
*Enterobacter sp.*	Standard	Standard	Single-room	Single-room	Contact; Single-room, if CPE
Other *Enterobacteriaceae*	Standard	Standard	Single-room	Single-room	Contact; Single-room, if CPE
*P. aeruginosa*	Single-room	Standard	Single-room	Single-room	Contact
*A. baumannii*	Single-room	Standard	Single-room	Single-room	Single-room with ante-room
*S. maltophilia*	Standard	Standard	Standard	Standard	Contact

German guideline: Each hospital defines local “High risk wards” (where infection and transmission of MDRO is highly critical and affects susceptible patients) or functional areas where special hygiene precautions for 3MRGN are performed. “Single-room” isolation comprises isolation of the patient in a separate room, but without dedicated staff. Persons entering a single-room have to wear disposable gowns; gloves should be worn during contact with the patient and the patient’s environment.

Dutch guideline: “Contact” precaution include wearing disposable gowns; gloves should be worn during contact with the patient and the patient’s environment. Many hospitals perform contact isolation in single-rooms; however this is not explicitly required by the guideline except for CPE (i.e. carbapenemase-producing organisms).

The German guideline advises single-room isolation of the patient on all wards of the hospital, whenever carriage or infection with a GN-MDRO classified as “4MRGN” is detected. For patients affected by GN-MDRO classified as “3MRGN” single-room isolation is required on high-risk wards only. Single-room isolation is always accompanied by wearing a disposable gown (when entering the room), gloves and, if indicated (e.g. tracheal suctioning), a surgical mask. Patients carrying 3MRGN can be seen with standard hygiene measures at non-high-risk wards. High risk-wards are defined by local infection control personnel in every hospital, but should comprise e.g. intensive care units, including neonatology and haemato-oncology wards according to the guideline.

In The Netherlands, isolation measures for “BRMO’s” are to a certain degree comparable, but show some differences. “Strict isolation” will be used for patients colonized or infected with a highly resistant *Acinetobacter spp.*. Strict isolation includes a single-room with ante-room, negative air pressure of the isolation room, wearing a disposable, long-sleeved gown, gloves and an FFP1 (filtering facepiece particle) mask. For the other GN-MDRO “contact isolation” is stipulated. Just like in Germany, this comprises wearing a disposable gown (before contact with the patient), gloves (when touching the patient and the patient’s immediate environment) and, if indicated (e.g. tracheal suctioning), a FFP1 mask. However, many hospitals in the Netherlands perform “contact isolation” in single-rooms (without ante-room) for ESBL-*Klebsiella* and Carbapenem resistant *Enterbacteriaceae* (CRE), despite the fact that single-room isolation is only “suggested” but not mandatory, according to the guideline.

According to the German guideline screening for 4MRGN carriage should be performed on all hospital wards for the following risk persons: 1.) contact with healthcare facilities in endemic countries; 2.) contact with a 4MRGN positive patient (same room). For these risk persons, pre-emptive isolation until the exclusion of 4MRGN is recommended. In the Netherlands, this policy of “search & isolate” is used for all GN-MDRO, has a few additional risk groups and is extended with a few Gram-positive MDRO, such as vancomycin-resistant enterococci and penicillin-resistant pneumococci. Consequently, these patients are screened and isolated until proven-negative, for all of the above mentioned MDROs.

The conclusions of the Workshop on “ESBL/Multidrug Resistant Gram-negatives (“MRGN”) harmonization of the new guidelines in Germany and The Netherlands” in Nijmegen are illustrated in Table 4. The experts indicated a strong need to share available data on the prevalence of GN-MDRO, especially in the border region. Additionally, missing data should be obtained by sentinel and research studies. Moreover, there was agreement on the need of harmonized guidelines for diagnostics and terminology. Finally, the experts consented that more research is needed to be able to estimate the costs of transmission and infection prevention measures with regard to multidrug-resistant microorganisms.

**Table 4:** **Actions in order to harmonize Dutch and German GN-MDRO guidelines according to the expert workshops**

**Workshop**	**Workshop**	**Workshop**
**Epidemiology**	**Diagnostics & Treatment**	**Infection Prevention**
1. Need for sharing and analyzing available data at the regional level.	1. Standardization and harmonization of diagnostics and sensitivity testing.	1. Further research on the spread of GN-MDRO is needed.
2. Agreements on data ownership are necessary.	2. Information regarding direct and indirect costs of transmission and control.	2. Research on spread of ESBL-producing strains in settings with and without selective digestive decontamination (SDD) is needed.
3. Standardize typing methods for GN-MDRO and perform ring trials for typing (e.g. next generation sequencing).	3. Further research on the impact of different GN-MDRO is needed.	3. Better training for healthcare workers with regard to basic hygiene.
4. Typing of all Carbapenem-resistant enterobacteria is necessary.		
5. A prevalence study on ESBL-producing enterobacteria is necessary.		
6. Cohort studies for GN-MDRO carriage over time.		

## Discussion

We identified that the Dutch and German guidelines show important discrepancies regarding the definition of GN-MDRO. In the Dutch guideline, defining GN-MDRO *Enterobacteriaceae* requires testing for certain mechanisms causing antibiotic resistance, i.e. ESBL and carbapenemase pheno- or genotypes have to be determined in line with routine microbiological diagnostics. The German guideline makes use of a newly created classification scheme (MRGN classification), based on phenotypic characterization and intended to guide infection control measures. This guideline is not meant to substitute reporting of resistance mechanisms detected by the laboratory, usually following the recommendations of EUCAST or CLSI standards. The MRGN classification grades multidrug-resistance by severity into 3MRGN and 4MRGN. For the correct classification as 3MRGN or 4MRGN, detection of specific resistance mechanisms like ESBL production is not required by the German guideline. The definition of GN-MDRO is mostly based on phenotypic susceptibility patterns, because the German guideline argues, that generally the transmission of resistant bacterial clones should be prevented whether the resistance mechanism is encoded on a plasmid or the chromosome. Detection of carbapenemase production, however, is essential, since according to the German guideline carbapenemase production in Enterobacteriaceae leads to classification as 4MRGN irrespective of other susceptibility test results.

A major advantage of testing samples for presence of defined resistance determinants is a better understanding of resistance spread, especially if resistance is conferred by mobile genetic elements. A disadvantage might be that it is difficult to cover all mechanisms causing multidrug resistance by this approach; exemplarily, chromosomally- or plasmid-encoded *ampC* is missed by the Dutch strategy.

Besides diagnostic issues, the main difference between the Dutch and German guideline is the divergent evaluation of ESBL-producing *Enterobacteriaeceae*. Whereas special hygiene measures are required for all patients with ESBL-producing enterobacteria in The Netherlands, the German guideline recommends special precautions only for those cases in which patients are colonized or infected with strains showing co-resistance to ciprofloxacin (“3MRGN”). This difference is relevant: In Germany, the national surveillance system for antibiotic resistance (https://ars.rki.de) has published that among *E. coli* derived from inpatients, 10.8% were overall resistant to third-generation cephalosporines (indicative for ESBL) [12] in 2012. Co-resistance of third-generation cephalosporines and ciprofloxacin occurred in 7.7% [13] of the *E. coli* isolates; i.e. 71% of third-generation cephalosporine-resistant *E. coli* isolates were ciprofloxacin resistant. In consequence, special hygiene precautions are estimated to be not applied for about 29% of patients with ESBL-producing *E. coli* in this example.

In addition, co-resistance to aminoglycosides and quinolones, which defines GN-MDRO among enterobacteria according to the Dutch guideline, is not considered in this context in hospitals on the German side of the border. This might be due to the fact that aminoglycosides are markedly less used in German than in Dutch hospitals.

In consequence, the fact that the definitions for GN-MDRO are different in the two current guidelines may lead to inefficient information transfer and different hygienic precaution measures when transferring a patient across the border. Therefore, the usage of a harmonized terminology would improve the possibilities for infection prevention, treatment and patient safety. Moreover, data on the prevalence of GN-MDRO in the border region should be reported in a way that they are directly comparable. Hence, the expert workshop revealed that harmonization of diagnostics report definitions is essential to facilitate cross-border cooperation between healthcare institutions. The initiation of cohort studies is warranted to yield comparable regional data.

With respect to recommendations on hygiene standards, both guidelines recommend single-room isolation of patients with carbapenem-resistant or carbapenemase-producing enterobacteria and *A. baumannii*. This is an important similarity of both guidelines. As the prevalence of carbapenem-resistance is still <1% for enterobacteria in both The Netherlands and Germany, infection control guidance should clearly focus on preventing dissemination of these GN-MDRO as transmission was recently observed in Italian healthcare facilities [14]. In this context, we also found major agreement of the two guidelines with respect to the definition of risk-patients for carriage of carbapenem-resistant *Enterobactericeae* and *Acinetobacter*, for whom screening should be considered. However, the need to ensure prevention of carbapenem-resistant *Enterobacteriaceae* and *Acinetobacter* in the border region is also underpinned by recent investigations indicating the more wide-spread [15] dissemination of these organisms in Germany compared to The Netherlands.

Regarding the use of special precautions for ESBL-*producing Enterobacteriaceae* the Dutch guideline is more “microbiology-driven” compared with the German one. If ESBL is detected, such precautions are recommended. The German guideline advises single-room isolation on high-risk wards, because until now there is no general evidence for a benefit from single room isolation for 3MRGN *E. coli* in endemic areas. But on high risk wards (e.g. intensive care units), ESBL-producing and ciprofloxacin-resistant *Enterobacteriaceae* are likely to be transmitted to patient groups susceptible to acquire infections rather than colonization with these GN-MDRO.

A clear problem related to these recommendations is that there is rising evidence that ESBL-producing *Enterobacteriaceae* including 3MRGN pathogens are already wide-spread in the general population. In The Netherlands ESBL-carriage was demonstrated in 8-10% of community patients [16], in Germany regional colonization was >6% [17]. Hence, risk-based screening may be misleading and isolation facilities might become rare in future. Many Dutch hospitals started to use different approaches for ESBL-producing *E. coli* and *Klebsiella*, due to the differences in their molecular epidemiology and spread; multi-clonal/rarely spreading versus monoclonal and spreading, respectively [18]. Even though much is known about the microbiology of GN-MDRO, more knowledge has to be gained on their transmission pathways, prevalence on admission and in the community, virulence or risk factors for acquisition in order to enable more tailored infection prevention guidance. In addition, the effectiveness of the treatment could be increased, if regional data on GN-MDRO were available at the bed-side.

The attendants of the workshop expressed that gathering regional data and data comparable between the two sides of the Dutch-German border is essential, because first, as a result of demographic changes in the border region, the proportion of population seeking medical treatment will rise including the proportion of those seeking treatment abroad. Second, particularly in the border region between two European high-income countries with cost-extensive healthcare systems, the number of highly susceptible individuals like neonates, immuno-compromised patients and elderly patients continues to increase due to implementation of advanced technologies. Together with an enhanced use of medical devices and implants vulnerable to bacterial contamination, hospital acquired infections are expected to rise, especially in the border region.

Development of new diagnostic technology for rapid identification and personalized therapy and infection prevention management seems also very relevant to the attendants.

Therefore, delivering innovative medical care and enabling “Healthy Ageing” will demand an increased level of infection control. This includes the necessity to cooperate across borders between countries (The Netherlands vs. Germany) as well as inter-sectorial borders (e.g. human vs. veterinary medicine; hospital vs. community healthcare) to a much larger extent than this is done nowadays. So far intersectorial cooperation has been focused on MRSA- management. The approach has to be stretched to all MDROs. The same holds for transborder cooperation in euregional networks including the public health structures (GGD/ÖGD) on both sides of the border. As previous studies performed within the Interreg IVa project EurSafety Health-net already demonstrated that synchronised strategies for screening of risk patients and standards of care in the framework of regional networks, were able to decrease MRSA prevalence [19], this shall inspire new projects in an Interreg V project called Health-i-care. This project in development, responds to an important finding of the published WHO report “Antimicrobial resistance: global report on surveillance 2014” saying that surveillance of antibacterial resistance is neither coordinated nor harmonized and there are many gaps in information on bacteria of major public health importance [20]. Health-i-care uses the existing cross-border network created by Mrsa-net and Eursafety Health-net and focuses on the innovative development of medical (E)-technology in collaboration between small medium enterprises (SME’s), universities and healthcare institutions of the border region for fostering patient safety, improving training and fostering sustainable healthcare.

## References

[1] Coque TM, Baquero F, Canton R (2008). Increasing Prevalence of ESBL-Producing Enterobacteraiceae in Europe. Eurosurveillance.

[2] Glasner C, Albiger B, Tambić Andrašević A, Canton R, Carmeli Y, Friedrich AW (2013). Carbapenemase-producing Enterobacteriaceaea in Europe: a survey among national experts from 39 countries, February 2013. Euro Surveill.

[3] Pallett A, Hand K (2010). Complicated urinary tract infections: practical solutions for the treatment of multiresistant Gram-negative bacteria. J Antimicrob Chemother.

[4] Peralta G, Sánchez MB, Garrido JC, De Benito I, Cano ME, Martínez-Martínez L (2007). Impact of antibiotic resistance and of adequate empirical antibiotic treatment in the prognosis of patients with Escherichia coli bacteraemia. J Antimicrob Chemother.

[5] Tumbarello M, Spanu T, Di Bidino R, Marchetti M, Ruggeri M, Trecarichi EM (2010). Costs of bloodstream infections caused by *Escherichia coli* and influence of extended-spectrum-β-lactamase production and inasequate initial antibiotic therapy. Antimicrob Agents Chemother.

[6] ECDC. Technical Report. The bacterial challenge: time to react. A call to narrow the gap between multidrug-resistant bacteria in the EU and the development of new antimicrobial agents. 2009. doi:10.2900/2518.

[7] Kennedy K, Collignon P (2010). Colonisation with Escherichia coli resistant to “critically important” antibiotics: a high risk for international travelers. Eur J Clin Microbiol Infect Dis.

[8] Rogers BA, Aminzadeh Z, Hayashi Y, Paterson DL (2011). Country-t-country transfer of patients and the risk of multi-resistant bacterial infection. Clin Infect Dis.

[9] KRINKO (2012). Hygienemaßnahmen bei Infektionen oder Besiedlung mit multiresistenten gramnegativen Stäbchen. Bundesgesundheitsblatt.

[10] WIP. Bijzonder resistente micro-organismen (BRMO). 2013.

[11] Ciccolini M, Donker T, Köck R, Mielke M, Hendrix R, Jurke A (2013). Infection prevention in a connected world: the case for a regional approach. Int J Med Microbiol.

[12] Resistenzübersicht. Antibiotika Resistenz Surveillance, Berlin. 2015. https://ars.rki.de/CommonReports/Resistenzuebersicht.aspx. Accessed 11 Feb 2015.

[13] Robert Koch-Institut. Ausgewählte gramnegative Erreger ausgewertet nach den KRINKO-Definitionen für Multiresistenz. 2015. https://ars.rki.de/Download/Multiresistenz/KRINKO/KRINKO_HO.pdf. Accessed 11 Feb 2015.

[14] Antimicrobial resistance interactive database. European Centre for Disease Prevention and Control, Stockholm. 2015. http://www.ecdc.europa.eu/en/healthtopics/antimicrobial_resistance/database/Pages/map_reports.aspx. Accessed 11 Feb 2015.

[15] European Centre for Disease Prevention and Control (2013). Carbapenemase-Producing Bacteria in Europe: Interim Results from the European Survey on Carbapenemase-Producing Enterobacteriaceae (EuSCAPE) Project.

[16] Reuland EA, Overdevest ITMA, al Naiemi N, Kalope JS, Rijnsburger MC, Raadsen SA (2012). High prevalence of ESBL-producing Enterobacteriaceae carriage in Dutch community patients with gastrointestinal complaints. Clin Microbiol Inf.

[17] Valenza G, Nickel S, Pfeifer Y, Eller C, Krupa E, Lehner-Reindl V (2014). Extended-spectrum-β-lactamase-producing escherichia coli as intestinal colonizers in the german community. Antimicrob Agents Chemother.

[18] Freeman JT, Nimmo J, Gregory E, Tiong A, De Almeida M, McAuliffe GN (2014). Predictors of hospital surface contamination with Extended-spectrum β-lactamase-producing *Escherichia coli* and *Klebsiella pneumoniae*: patient and organism factors. Antimicrob Resist Infect Control.

[19] Jurke A, Köck R, Becker K, Thole S, Hendrix R, Rossen J (2013). Molecular epidemiology of methicillin-resistant Staphylococcus aureus (MRSA): think regionally but use globally uniform typing languages. Euro Surveill.

[20] World Health Organization. Antimicrobial resistance: global report on surveillance. 2014.

